# Achievements hitherto and a new editorial team for the *South African Journal of Psychiatry*

**DOI:** 10.4102/sajpsychiatry.v23i0.1145

**Published:** 2017-07-15

**Authors:** Werdie C.W. van Staden

**Affiliations:** 1 Department of Psychiatry, Faculty of Health Sciences, University of Pretoria, South Africa

The *South African Journal of Psychiatry* (SAJP) is in its 24th year of publishing scholarly work in psychiatry. This is no small achievement. The archives of all the articles that have been published (all from 2002 are available open access at www.sajp.org.za) is evidence of this as well as the various milestones that have been achieved over the years in the face of various daunting challenges along the way.

I remember clearly the discussion led by Prof. Robin Emsley in the early 1990s on a SASOP national executive meeting (I was an observer as a psychiatry registrar at the time) where he was pioneering the route for establishing the SAJP. Prof. Cliff Allwood and Prof. Robin Emsley took the reign consecutively for the first 12 years, and I have been carrying the editor-in-chief baton for the past 11 years, supported from 2013 by three deputy editors, namely, Prof. Liezl Koen, Prof. Soraya Seedat and Prof. Bernard Janse van Rensburg.

Over the past decade, SAJP has achieved a gradual increase in the quality and number of articles published, featuring work by researchers from South Africa, the rest of Africa and increasingly from other continents. From processing half a dozen articles at a given time 10 years ago, the editorial task has reached recently about 70 submissions at a given time, for which each requires back and forth correspondence with its authors, reviewers and the copy editors. Much happens behind the scenes, including guidance and finding ways in which a potentially worthy article can be rescued when, for example, it is poorly written, dealing with (eminent) authors who feel affronted, etc. Emerging from these efforts, 30% – 40% of all submissions during the past 3 years have resulted in published articles. All this has been paying off. The expectations set in 2006,^1,2^ I believe, have been met owing to excellence in research and publishing afforded by all the role-players.

Other than the contents of SAJP, the most striking milestones during the past decade are the various international listings or indexing that have been awarded, including some of the most prestigious in the world. These listings are on Web of Science Core Collection, Science Citation Index Expanded, SCIE (previously known as ISI); Web of Science Core Collection, Social Sciences Citation Index, SSCI (previously known as ISI); SCOPUS; GALE; CENGAGE Learning; SciELO SA; African Journals Online (AJOL); African Index Medicus; Google Scholar; Norwegian Register for Scientific Journals, Series and Publishers, Level 1.

Other milestones during the past decade include the transition from a manual submission and communication process to a web-based software platform, having new and past issues available gratis on the web and open access to all interested readers, and the transition to the current publishing house (AOSIS). A major challenge arose during 2014, when the previous publishing house had unilaterally enforced a number of changes including the defiance of contractual arrangements for publishing SAJP, terminating the website and editorial software platform dedicated exclusively for SAJP and replacing this infrastructure with a website shared with other journals. Adding insult to injury, the new website crashed irreversibly, without the publisher having secured adequate backup of content. Consequently, several submissions and communiques were lost, which then had to be recovered through external means insofar as possible. As the relationship with the publisher had become untenable, the SASOP Board of Directors then decided to contract with another. The resulting transition in the beginning of 2015 was successful but some damage was inevitable as seen, for example, in the subsequent drop in the journal’s impact factor. Fortunately, preliminary indications are that the impact factor is on the rise again. Moreover, downloads of full articles currently reach close to 3000 per month.

SAJP has survived these trying times and is flourishing in quality and quantity more so than ever before. Its growth and the rapidly changing world of scientific publications bring about higher demands editorially and for its sustenance. The SASOP Board of Directors has recognised these challenges, by which henceforth the contracted editor-in-chief will be receiving a stipend for services and will no longer be tasked with the financial and funding matters of the journal. These developments should provide for gearing up to a next level of accomplishments.

**FIGURE 1:**
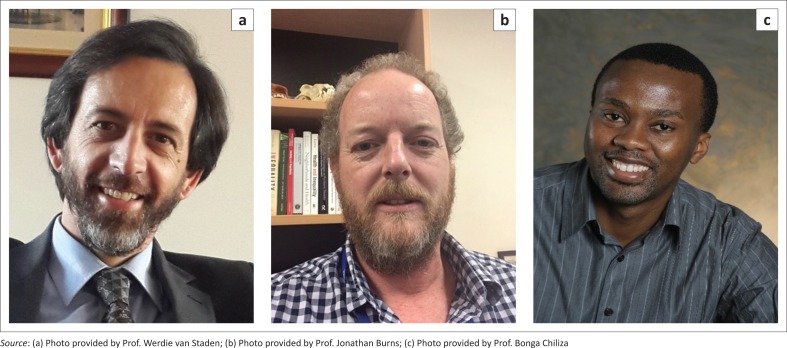
(a) Prof. Werdie van Staden Outgoing Editor-in-Chief (b) Prof. Jonathan Burns New Editor-in-Chief (c) Prof. Bonga Chiliza New Deputy Editor.

The SASOP Board of Directors, furthermore, upon my request almost a year ago, has secured a new editorial team as from July 2017: Prof. Jonathan Burns from the University of Exeter as editor-in-chief, Prof. Bonga Chiliza (University of KwaZulu-Natal) as deputy editor and Prof. Bernard Janse van Rensburg (University of the Witwatersrand), Soraya Seedat (Stellenbosch University), Christopher Szabo (University of the Witwatersrand) and myself (University of Pretoria) as associate editors.

I attest that editorship of SAJP has taken a lot of courage (and work, of course). I sincerely congratulate our colleagues on being assigned to their new roles and wish them abundant success with meeting the ever-increasing demands and seeking out creatively further ‘gear changes’ for SAJP. I implore all psychiatrists in their various roles as well as other stakeholders to support actively the new editorial team in every sensible way.
